# Patient reported outcome following incisional hernia repair: A survey on 163 patients at two maximum care hospitals

**DOI:** 10.1016/j.amsu.2019.06.005

**Published:** 2019-06-15

**Authors:** Christoph Paasch, Eric Lorenz, Stefan Anders, Gianluca De Santo, Katherina Boettge, Ulrich Gauger, Roland Croner, Martin W. Strik

**Affiliations:** a Department of General, Visceral and Cancer Surgery, Helios Klinikum Berlin-Buch, Berlin, Schwanebecker Chaussee 50, 13125, Berlin, Germany; b Department of General, Abdominal and Vascular Surgery, University Hospital, Magdeburg, Leipziger Str. 44, 39120, Magdeburg, Germany; c No Insurance Surgery, 9121 W Russell Rd Ste 115, 89148, Las Vegas, United States; d Private Statistical Consultant, Germany

**Keywords:** Abdominal binder, Open incisional hernia repair, Physical rest, Postoperative pain, Return to work

## Abstract

**Introduction:**

Incisional hernias of the abdominal wall are frequent complications after laparotomy (9–20%) and often need incisional hernia repair (IHR). In order to ensure wound healing and to therefore prevent postoperative short and long term morbidity carrying an abdominal binder (AB) and physical rest is frequently advised. However, there is a lack of evidence concerning clinical effects regarding these recommendations. Hence, we conducted a survey to analyze the patient reported outcome following IHR.

**Methods:**

From December 2017 to May 2018, we conducted a survey among 270 patients who underwent open and laparoscopic IHR at two maximum care hospitals. They were interviewed about their type of operation, postoperative treatment, recommendations, and outcome.

**Results:**

163 patients replied to the questionnaire. The average age was 63.2 ± 12 years. 74 patients were female and 89 were male. 32.6% of the patients reported an AB-induced immobility and 71.2% reported that the AB reduced pain after IHR. A prolonged period of physical rest and the use of an AB had no statistical significance on postoperative morbidity.

**Conclusions:**

Due to our findings we assume that the AB may induce immobility and reduce postoperative pain. A prolonged period of physical rest and wearing an AB does not seem to have an impact on the postoperative outcome following IHR. Therefore, a shortened duration of physical rest and wearing an AB following IHR should be taken under consideration. To reveal more evidence on this topic further clinical trials are essential.

## Highlights

• The use of an abdominal binder may reduce pain following incisional hernia repair.
• On third of the questioned patients reported, that the AB induced immobility.
• A prolonged period of physical rest and carrying an abdominal binder may not prevent recurrences.

## 1 Background

Incisional hernia is a common complication following abdominal surgery with an incidence of 9–20% [1]. Diabetes mellitus, obesity, coronary heart disease, smoking, and advanced age are described as risk factors for the development of incisional hernias [1,2]. These hernias frequently lead to chronic pain, respiratory dysfunction, discomfort, immobility, strangulation and incarceration. In these cases, hernia repair must be performed [3].

The recurrence rate with a mesh is lower and therefore it is strongly recommended when IHR is performed [2,4]. In addition, the International Endohernia Society (EHS) proposes that when a hernia has a gap of more than two cm in diameter, the placement of a mesh is necessary [3,5,6].

There is a large number of different surgical techniques varying both in their operative approach and in their positioning of mesh [7]. In case of recurrences, prolonged wound pain and infection, the open IHR with sublay or onlay mesh placement, as well as the laparoscopic intraperitoneal onlay mesh (IPOM) repair are described as being sufficient approaches for IHR [[8], [9], [10]].

Frequently in Western Europe, surgical departments are prescribing an AB and recommending physical rest as postoperative treatment following an IHR [[11], [12], [13]]. The AB shall reduce pain, the rate of seroma formation, and prevent early postoperative damages, such as hernia recurrence or damage to the mesh sutures. Moreover, in order to ensure the healing of the fascia and thereby preventing relapses, physical rest for several weeks postoperatively with the avoidance of heavy lifting is advised [11,12]. However, there is a lack of evidence concerning clinical effects of ABs and physical rest following an IHR. Therefore, postoperative recommendations of surgical departments all over Germany seem to be random, as investigated by our workgroup [11]. A shortened period of physical rest and wearing an AB may have a social-economic impact by reducing the individual capacity for work and mobility. Therefore, further investigations are necessary.

Due to the lack of evidence in postoperative treatment, we conducted a survey among 270 patients who underwent open and/or laparoscopic IHR.

## 2 Methods

From December 2017 to May 2018, we conducted a survey on 270 patients who underwent open and/or laparoscopic IHR in the Department of General, Visceral, and Cancer Surgery at Helios Klinikum Berlin-Buch (Germany) or in the Department of General, Abdominal, Vascular and Transplant Surgery at the University Hospital, Magdeburg (Germany). The patients were operated on between March 2013 and December 2017.

In November 2017, the questionnaire was developed and designed by the authors of both departments. The questionnaire was sent out by mail. The patients were asked to complete the questionnaires anonymously and were advised to send them back to the hospital where they underwent the surgical procedure.

They were surveyed about their profession, age, gender, body mass index (BMI), the recommended time of physical rest (weeks), the actual time of physical rest (weeks), the recommended time of wearing an AB (in weeks), the actual time of wearing an AB (weeks). The patients were also asked about the postoperative incidence of hernia recurrence (yes/no), seroma formation (yes/no), pulmonary infection (yes/no), pain reducing effects of the AB (yes/no) and movement limitation induced by the AB after surgery (yes/no) (Table 1).

**Table 1:** Summarized survey data.

Variable		n = 163
Laparoscopical IHR		80
Open IHR		73
Age	years	63,2 ± 12
Gender	male	74
	female	89
BMI	<25	31
	25–30	67
	>30	60
Pensioners		90
Invalids, Unemployed		10
Working individual		63
Average duration of physical rest	weeks	6,2 ± 4,2
Average duration of carrying an AB	weeks	7,6 ± 4,2
Hernia relapse		25
Seroma formation		14
Pulmonary infection		15
AB induced immobility		50
AB induced reduction of pain		115

AB: Abdominal binder; BMI: body mass index; IHR: Incisional hernia repair.

We define physical rest as a state of low activity, which includes the avoidance of lifting of heavy weight.

### 2.1 Inclusionary criteria

Patients undergoing open and laparoscopic incisional hernia repair were included.

### 2.2 Exclusionary criteria

Patients who answered the questionnaire who were not anonymous were excluded.

### 2.3 Surgical approach

All patients gave informed consent prior to their operation. The surgical procedure consisted of the laparoscopic intraperitoneal onlay-mesh (IPOM) repair, open IPOM, open onlay or open sublay mesh placement. All patients received preoperative antibiotic prophylaxis with Cefuroxime and in cases of penicillin allergy they received Ciprofloxacin.

### 2.4 Postoperative recommendation

A physical rest of 4–6 weeks and wearing an AB during daytime for the same period was advised following an open IHR.

A physical rest of 4–6 weeks and wearing an AB during the daytime for the same period was advised following laparoscopic IHR.

The Department of General, Visceral and Cancer Surgery of the Helios Klinikum Berlin-Buch prescribed the AB named ‘Verba^®^’ (Company: PAUL HARTMANN AG).

The Department of General, Abdominal, Vascular and Transplant Surgery of the University Hospital, Magdeburg prescribed the AB named ‘HELIOS Leibbandage Economy^®^’ (Company: med.kontex GmbH).

### 2.5 Univariate analysis

For more detailed analyses we subdivided the cohort of all patients into the following subgroups:

#### 2.5.1 Abdominal binder

Group I consists of patients who wore the AB.
Group II consists of patients who did not wear the AB (Table 2).

**Table 2:** Univariate analysis of the abdominal binders on incisional hernia repair.

Variable		No Abdominal binder n = 17	Abdominal binder n = 146	p value
Laparoscopic IHR		14	66	0,003
Open IHR		2	71	
Age	years	63,6 ± 9,9	63,2 ± 12	0,814
Gender	female	9	65	0,6095
	male	8	81	
BMI	<25	4	29	0,946
	25–30	8	57	
	>30	5	55	
Average duration of physical rest	weeks	3,9 ± 2,7	6,5 ± 4,2	0.1302
Hernia relapse		1	24	0,4676
Seroma formation		0	14	0.3637
Pulmonary infection		0	15	0.999

IHR: Incisional hernia repair.

#### 2.5.2 Abdominal binder; age related

Depending on median age of the questioned patients (>median; <median) two analysis were conducted.

a)
  Group I consist of patients (<median age)) who wore the AB
  Group II consist of patients (<median age)) who did not wear the AB.
b)
  Group I consist of patients (>median age)) who wore the AB.
  Group II consist of patients (>median age)) who did not wear the AB.

#### 2.5.3 Duration of physical rest

Group I consists of patients with a postoperative duration of physical rest between 0 and 4 weeks.
Group II consists of patients with a postoperative duration of physical rest between 5 and >14 weeks (Table 3).

**Table 3:** Univariate analysis of survey data among indiviuduals with different durations of physical rest.

Variable		Group I n = 33	Group II n = 46	p value
Laparoscopical IHR		26	23	0.0084
Open IHR		6	22	
Age	year	61,8 ± 10	52,9 ± 11,1	0.0037
Gender	female	12	19	0.8156
	male	21	27	
BMI	<25	7	3	0.4482
	25–30	10	18	
	>30	16	20	
Abdominal binder	weeks			
average duration of carrying		5,8 ± 4,1	8,3 ± 4,4	0.0158
carried less than recommended		2	7	0.5904
carried like recommended		13	18	
carried longer than recommended		8	14	
No Abdominal binder		5	4	0.4792
Hernia relapse		3	4	0.9999
Seroma formation		1	4	0.6427
Pulmonary infection		1	4	0.6427
Abdominal binder induced immobility		10	18	0.4627
Abdominal binder induced reduction of pain		25	33	0.3379

Group I consists of patients with postoperative duration of physical rest between 0 and 4 weeks.

Group II consists of patients with postoperative duration of physical rest between 5 and >14 weeks.

IHR: Incisional hernia repair.

#### 2.5.4 Load-dependent work according to the definition by Rohmert and Rutenfranz 1983 [12].bib12

Group I consists of professions with light and heavy manual labor with hands and arms.
Group II consists of professions with light, heavy, and very heavy physical labor (Table 4).

**Table 4:** Univariate analysis of survey data among indiviuduals with different load-depended work.

Variable		Group I n = 30	Group II n = 33	p value
Laparoscopical IHR		17	22	0.7897
Open IHR		11	11	
Age	year	59 ± 9,6	51,8 ± 12,4	0.0185
Gender	*female*	14	13	0.6164
	male	16	20	
BMI	<*25*	4	7	0.7485
	25–30	11	12	
	>*30*	14	13	
Physical rest	weeks			
average duration		5,6 ± 2,5	7,8 ± 4,4	0.1006
less than recommended		4	4	0.7719
like recommended		9	14	
longer than recommended		6	6	
Abdominal binder	weeks			
average duration of carrying		6,7 ± 4,1	8,5 ± 4,2	0.1280
carried less than recommended		5	2	0.0767
carried like recommended		7	13	
carried longer than recommended		14	9	
No Abdominal binder		2	2	0.9999
Hernia relapse		5	1	0.0933
Seroma formation		2	3	0.9999
Pulmonary infection		2	3	0.9999
Abdominal binder induced immobility		8	17	0.0667
Abdominal binder induced reduction of pain		25	25	0.4770

Group I consisted of professions w/light and heavy manual labor w/hands and arms.

Group II consisted of professions w/light, heavy and very heavy physical labor.

IHR: Incisional hernia repair.

#### 2.5.5 Surgical approach

Group I consists of patients who underwent open IHR.
Group II consists of patients who underwent laparoscopic IHR (Table 5).

**Table 5:** Univariate analysis of survey data's concerning the surgical approach.

Variable		Open hernia repair n = 73	Laparoscopic hernia repair n = 80	p value
Physical rest	weeks			
average duration		8,2 ± 4,3	5,2 ± 3,8	0.0009
less than recommended		2	8	0.4899
like recommended		14	22	
longer than recommended		3	10	
Abdominal binder	weeks			
average duration of carrying		9 ± 4,1	6,2 ± 4,2	<0.0001
carried less than recommended		8	4	0.5515
carried like recommended		28	30	
carried longer than recommended		20	18	
No Abdominal binder		2	14	0,0030
Abdominal binder induced immobility		30	20	0.2129
Abdominal binder induced reduction of pain		54	55	0.2505

### 2.6 Statistics

The results of the questionnaire were entered into Microsoft^©^ Excel. The statistical analysis was done by using R 3.5.0.

In order to compare the baseline data (age, gender, BMI) and outcome between the groups as defined above, the mean, the standard deviation, the minimum, the maximum, and the median were calculated for continuous variables. For categorical variables, cross-tables were calculated.

The normality of continuous variables was tested with the Shapiro-Wilk-test. In the case of normally distributed continuous variables, t-tests for independent groups were used to compare groups, otherwise Wilcoxon-tests were used. In the case of categorical variables, Fishers exact test was used. A p-value of less than 0.05 was regarded as statistically significant.

For all analyses only available data was used, no imputation of missing values was done. Hence for some variables less than 163 data points were available.

Since this is an exploratory study, no adjustment of the type-I error was done.

The work has been reported in line with the STROCSS criteria [14].

### Ethical approval

The survey at hand was approved by the medical ethical committee of the Otto von Guericke University in Magdeburg, Germany.

#### 2.6.1 Study registration

The study has been registerd in the german clinical trial registry (DRKS, Deutsches Register klinischer Studien; https://www.drks.de/) with the ID DRKS00016634.

## 3 Results

163 patients applied the questionnaire anonymously (Berlin: n = 115; Magdeburg: n = 48).

### 3.1 Baseline characteristics

73 (47.7%) patients underwent open IHR. 80 (52.3%) patients were operated on laparoscopically. 10 patients did not applied the questionnaire in terms of their surgical approach. The average age was 63.2 ± 12 years. 74 (45.4%) patients were female and 89 (54.6%) patients were male. 31 (19.6%) patients had a BMI below 25 kg/m^2^, 67 (41.1%) between 25 and 30 kg/m^2^ and 60 (36.8%) above 30 kg/m^2^ (Table 1).

#### 3.1.1 Patients professions

7 patients did not answer the question concerning their professions. 90 patients were retired. Two patients were invalid patients and one individual (0.6%) was unemployed at the time of the survey conduction. 63 patients were working, when they replied to the questionnaire.

### 3.2 Postoperative recommendations

79 patients answered the questions regarding physical rest after surgery. The average duration of physical rest was 6.2 ± 4.2 weeks. 6 patients did not comply with physical rest postoperatively. 7 Individuals had physical rest for two weeks, 10 patients had physical rest for three weeks, 10 patients had physical rest for 4 weeks, 5 patients had physical rest for 10 weeks, 9 patients had physical rest for 6 weeks, two patients had physical rest for 7 weeks, 7 patients had physical rest for 8 weeks, two patients had physical rest for 10 weeks, 5 patients had physical rest for 12 weeks, 1 patient had physical rest for 13 weeks, and 10 patients had physical rest for 14 weeks or more than 14 weeks. 61 patients answered the questions concerning conducted and advised physical rest. 13 (21.1%) patients physically rested less than recommended, 37 (60.7%) rested as recommended, and 11 (18.1%) rested longer than recommended.

163 patients replied to the questionnaire concerning the use of an AB. 17 patients did not use the AB and 146 patients used an AB after surgery. The average duration of wearing an AB was 7.6 ± 4.2 weeks. 132 patients answered the question for how many weeks they wore an AB.

113 patients answered the question concerning conducted and advised carrying of an AB. 13 (11.5%) patients wore the AB less than the amount of time recommended, 61 (54%) wore the AB as recommended, and 39 (34.5%) wore the AB longer than recommended (Table 1).

### 3.3 Postoperative outcome

Among the 163 analyzed individuals, 25 (15.3%) patients suffered from a relapse, 14 (8.6%) from a seroma formation and 15 (9.2%) from a pulmonary infection. 50 (34.2%) patients reported of immobility caused by the AB. 115 (78.8%) patients reported that the AB reduced postoperative pain (Table 1).

### 3.4 Univariate analysis of the abdominal binders on incisional hernia repair

17 patients did not wear an AB. 146 used an AB.

In the “No Abdominal binder” group, 14 patients were operated on through a laparoscopic technique IHR and two (12.5%) patients received open IHR. The average age was 63.6 ± 9.9 years. 9 patients were female and 8 were male. 4 patients had a BMI below 25 kg/m^2^, 8 between 25 and 30 kg/m^2^, and 5 above 30 kg/m^2^. The average duration of physical rest was 3.9 ± 2.7 weeks. One patient suffered from a hernia recurrence. No patient suffered from a seroma formation or a pulmonary infection.

In the “Abdominal binder” group, 66 patients were operated on through a laparoscopic technique and 71 patients were operated on through an open technique. The average age was 63.2 ± 12 years. 65 patients were female and 81 were male. 29 patients had a BMI below 25 kg/m^2^, 57 between 25 and 30 kg/m^2^, and 55 above 30 kg/m^2^. The average duration of physical rest was 6.5 ± 4.2 weeks. 24 patients suffered from a hernia recurrence. 14 patients suffered from a seroma formation and 15 individuals suffered from a pulmonary infection.

Regarding the average duration of physical rest (p = 0.1302), the postoperative incidence of pulmonary infections (p = 0.9999), relapses (p = 0.4676), and seroma formations (p = 0.3637), no statistically significant differences were detected between the two groups after univariate analysis. Concerning the surgical approaches, a statistical significance with p = 0.003 was revealed (Table 2).

### 3.5 Age related univariate analysis of the abdominal binders on incisional hernia repair

#### 3.5.1 Two univariate analysis were conducted related to median age (64 years)

Concerning patients below median age 5 patients did not wear an AB. 68 used an AB.

In the “No Abdominal binder” group, 4 patients were operated on through a laparoscopic technique IHR and no patients received open IHR. The average age was 52.8 ± 7.98 years. 3 patients were female and 2 were male. The average BMI was 33.6 ±(7.10). No patient suffered from a seroma formation or a relapse.

In the “Abdominal binder” group, 34 patients were operated on through a laparoscopic technique and 30 patients were operated on through an open technique. The average age was 54.6 ± 9.3 years. 27 patients were female and 41 were male. The average BMI was 30.2 ± (6.34). 6 patients suffered from a hernia recurrence. 7 patients suffered from a seroma formation. 24 patients (35.8%) reported that the AB limitated mobilization. 54 patients reported that the AB reduced pain.

Regarding biometric baselines (age: p = 0.646; gender: p = 0.396; BMI: p = 0.351), treatment (p = 0.124) and outcome (seroma and relapse p = 1.000) no statistical significance was revealed.

Concerning patients above median age 5 patients did not wear an AB. 68 used an AB.

In the “No Abdominal binder” group, 6 patients were operated on through a laparoscopic technique IHR and 2 patients received open IHR. The average age was 70.4 ± 2.92 years. 4 patients were female and 4 were male. The average BMI was 29.1 ±(4.65). No patient suffered from a seroma formation. One patient suffered from a relapse.

In the “Abdominal binder” group, 22 patients were operated on through a laparoscopic technique and 34 patients were operated on through an open technique. The average age was 73.2 ± 6.1 years. 28 patients were female and 30 were male. The average BMI was 28.6 ± (5.59). 13 patients suffered from a hernia recurrence. 6 patients suffered from a seroma formation. 18 patients (32.1%) reported that the AB limitated mobilization. 45 patients (86.5%) reported that the AB reduced pain.

Regarding biometric baselines gender (p = 1.000) and BMI (p = 0.797), treatment (p = 0.124) and outcome (seroma and relapse p = 1.000) no statistical significance was revealed. The groups statistical significant differed in terms of age (p = 0.042).

### 3.6 Univariate analysis of survey data among individuals with different durations of physical rest

Group I (n = 33) consists of patients with a postoperative duration of physical rest between 0 and 4 weeks. 26 patients were operated on through a laparoscopic technique and 6 patients were operated on through an open technique. The average age was 61.8 ± 10 years. 12 patients were female and 21 were male. 7 patients had a BMI below 25 kg/m^2^, 11 between 25 and 30 kg/m^2^, and 15 above 30 kg/m^2^. 33 patients replied to the questionnaire concerning the use of an AB. 5 patient did not use an AB, and 28 patients used an AB after surgery. The average duration of wearing an AB was 5.8 ± 4.1 weeks. Two (8.7%) patients wore the AB less than recommended, 12 (52.2%) wore the AB as recommended, and 8 (34.8%) wore the AB longer than recommended. Three patients suffered from hernia recurrence, One patient suffered from a seroma formation, and two patients suffered from a pulmonary infection. 10 individuals reported that the AB induced immobility. 25 patients reported that the AB reduced pain.

Group II (n = 46) consists of patients with a postoperative duration of physical rest between 5 and >14 weeks. 23 patients were operated on through a laparoscopic technique and 22 patients were operated on through an open technique. The average age was 52.9 ± 11.1 years. 19 patients were female and 27 were male. 5 patients had a BMI below 25 kg/m^2^, 18 between 25 and 30 kg/m^2^, and 20 above 30 kg/m^2^. 46 patients replied to the questionnaire concerning the use of an AB. 42 patients used an AB after surgery. The average duration of wearing an AB was 8.3 ± 4.4 weeks. 7 patients carried the AB less than recommended, 18 as recommended and 14 longer than recommended. 4 patients suffered from a hernia recurrence, 4 patients suffered from a seroma formation, and 4 patients suffered from a pulmonary infection. 18 individuals reported that the AB induced immobility. 33 patients reported that the AB reduced pain.

Regarding the postoperative incidence of pulmonary infections, (p = 0.6427), relapses (p = 0.999), and seroma formations, (p = 0.6427) no statistically significant differences were detected between the two groups following univariate analysis.

Concerning the surgical approach, (p = 0.0084), the age (p = 0.0037), and the average duration of carrying an AB, (p = 0.0158), a statistical significance was revealed (Table 3).

### 3.7 Univariate analysis of survey data among individuals with different load-dependent work

The group of working patients (n = 63), was further differentiated into two subgroups (methods page 2). Among the patients of group I (light and heavy manual labor with hands and arms; n = 30), are two authors, one banker, one bookkeeper, four clerks, one client advisor, one construction manager, two drivers, two engineers, four employees on field staff, one grinder, one journalist, three office employees, one hairdresser, one market manager, one medical laboratory scientist, one merchant, one social economist, one social worker, and one watchmaker. 17 patients were operated on through a laparoscopic technique and 11 patients were operated on through an open technique. The average age was 59 ± 9.6 years. 14 patients were female and 16 were male. 4 patients had a BMI below 25 kg/m^2^, 12 between 25 and 30 kg/m^2^, and 13 above 30 kg/m^2^. The mean duration of physical rest was 5.6 ± 2.5 weeks. 4 patients physically rested for less than the recommended duration, 8 rested as recommended, and 6 rested longer than recommended. Of the 30 patients who replied to the questionnaire, 28 patients wore an AB postoperatively. Two patients did not use an AB The mean duration of carrying an AB was 6.7 ± 4.1 weeks. 26 patients indicated how long they used the AB. 5 patients (19.2%) wore the AB less than recommended, 7 (26,9%) wore the AB as recommended, and 14 (53.8%) wore the AB longer than recommended. 5 patients suffered from a hernia recurrence, two patients suffered from a seroma formation, and two patients suffered from a pulmonary infection. 8 individuals reported that the AB induced immobility. 25 patients reported that the AB reduced pain.

Among the patients of group II (light, heavy and very heavy physical labor; n = 33), are two butchers, two caretakers, one cleaner, one construction worker, four cooks, one farmer, two housewives, one kindergarten teacher, one locksmith, nine mechanics, four nurses, three sellers, and two storekeepers. 22 patients were operated on through a laparoscopic technique and 11 patients were operated on through an open technique. The average age was 51.8 ± 12.4 years. 13 patients were female and 20 were male. 7 patients had a BMI below 25 kg/m^2^, 12 between 25 and 30 kg/m^2^ and 14 above 30 kg/m^2^. The average duration of physical rest was 7.8 ± 4.4 weeks. 4 patients physically rested less than the recommended duration, 14 rested as recommended, and 6 rested longer than recommended. 33 patients replied to the questionnaire regarding the use of an AB. 31 patients used an AB after surgery. The mean duration of wearing an AB was 8.5 ± 4.2 weeks. Two patients did not use an AB. 2 patients wore the AB less than recommended, 13 wore the AB as recommended, and 9 wore it longer than recommended. One Patient suffered from a hernia recurrence, three patients suffered from a seroma formation, and three patients suffered from a pulmonary infection. 17 individuals reported that the AB induced immobility. 25 patients reported that the AB reduced pain.

In terms of the surgical approach (p = 0.7897), gender (p = 0.6164), BMI (p = 0.7485), duration of postoperative physical rest (p = 0.7719), duration of wearing the AB (p = 0.0767), AB induced immobility (p = 0.0667), AB induced reduction of postoperative pain (p = 0.4770) and postoperative morbidity such as incidence of pulmonary infections (p = 0.999), recurrences (p = 0.093), seroma formations (p = 9999), AB induced immobility (p = 0.0667), and reduction of pain (p = 0.4770), no statistical significant differences were detected between the two groups after univariate analysis.

Concerning the age a statistical significance with p = 0.0185 was calculated (Table 4).

### 3.8 Univariate analysis of survey data concerning the surgical approach

10 patients were excluded as they did not answer which surgical technique was performed.

Among the patients who underwent open IHR (n = 73), the average age was 64.3 ± 10.4 years. 31 patients were female and 42 were male. 15 patients had a BMI below 25 kg/m^2^, 21 between 25 and 30 kg/m^2^, and 33 above 30 kg/m^2^. Among the patients who were operated on through IPOM-technique (n = 80), the average age was 61.8 ± 13.7 years. 39 patients were female and 41 were male. 16 patients had a BMI below 25 kg/m^2^, 31 between 25 and 30 kg/m^2^, and 32 above 30 kg/m^2^.

Regarding the age (p = 0.2419), the gender (p = 0.5162), and the BMI (p = 0.5276), no statistical significant differences were detected between the two groups after univariate analysis.

19 patients answered the questions concerning advised and actually conducted physical rest after surgery. The average duration of physical rest was 8.2 ± 4.2 weeks. Two patients physically rested less than recommended, 14 rested as recommended, and three rested for longer than recommended. 73 patients replied to the questionnaire concerning the use of an AB. Two patients did not use the AB. The average duration of wearing an AB was 9 ± 4.1 weeks. 8 patients wore the AB for less than the recommended duration, 28 wore the AB for the duration recommended, and 20 wore the AB for longer than recommended.

Among the patients who underwent laparoscopic IHR (n = 80), 5 patients suffered from a relapse, 6 from a seroma formation and 8 from a pulmonary infection. 20 patients reported that the AB induced immobility. 55 patients reported that the AB reduced pain after surgery. 40 patients answered the questions concerning physical rest after surgery. The average duration of physical rest was 5.2 ± 3.8 weeks. 8 patients physically rested less than recommended, 22 patients rested as recommended, and 10 patients rested longer than recommended. 80 patients replied to the questionnaire concerning the use of an AB. 14 patients did not use the AB after surgery. The average duration of wearing an AB was 6.2 ± 4.2 weeks. 4 patients wore the AB less than recommended, 30 wore the AB as recommended, and 18 wore the AB longer than recommended. Among the patients who underwent open IHR (n = 73) 16 patients had a recurrence, 8 patients had a seroma formation, and 6 had a pulmonary infection. 30 patients reported that the AB induced immobility. 54 patients reported that the AB reduced pain following IHRRegarding the recommendations of physical rest (p = 0.4899), AB induced immobility (p = 0.2129), and the reduction of pain (p = 0.2505), no statistical significant differences were detected between the two groups.

Concerning the avoidance of wearing an AB (p = 0.0030), the average duration of physical rest (p = 0.0009), wearing an AB (p= <0.0001) and relapse rate (p = 0.0085), a statistical significance was revealed (Table 5).

## 4 Discussion

### 4.1 Abdominal binder

After laparotomies in Western Europe, it is often advised to wear an AB to reduce the rate of seroma formations, pain, to facilitate wound healing, and thereby to prevent recurrences [11,12]. A survey that was conducted by Bouvier et al. among French surgeons revealed that 94% of 50 surgical departments were prescribing an AB following laparotomies in France. The expected benefit was the prevention of abdominal-wall complications [12]. In addition, we performed a survey among surgical departments in Germany. As a result, 70.5% of the surgical departments in Germany prescribed an AB as postoperative treatment [11]. Throughout literature, the evidence for wearing an AB is low. Rothman et al. conducted a systematic review and revealed a poor quality of the literature on this topic. The authors suggested that wearing an AB after a major abdominal surgery may reduce pain and improve physical function [13]. To our knowledge in terms of wearing ABs after hernia repair, only two relevant articles were published. Christoffersen et al. performed a randomized clinical trial (RCT) on the postoperative use of an AB after laparoscopic umbilical and epigastric hernia repair. Data from 56 patients (AB, n = 28; no binder, n = 28) were analyzed [15]. No significant effects on pain, movement limitations, seroma formation, fatigue, general well-being, or quality of life were revealed. Kaafarani et al. conducted a RCT of 145 patients undergoing open and laparoscopic IHR to investigate factors leading to seroma formation. The trial revealed that the duration of wearing an abdominal binder had no statistical significant impact on seroma formation [16].

Due to the lack of evidence concerning IHR, we performed a survey among German surgical departments. No correlation between the prolonged wearing of an AB and the recurrence rate was revealed [11]. Additionally, we conducted the survey at hand among 163 patients undergoing IHR. Possible positive effects of the AB were analyzed. No statistical significant differences were detected between the AB group (n = 146) and the non-AB group (n = 17) after univariate analysis (Table 4) regarding the postoperative incidence of pulmonary infections (p = 0.9999), recurrences (p = 0.4676), and seroma formations (p = 0.3637). In addition we further univariate analyzed the possible effects of the AB comparing patients above and below median age (64 years). In terms of biometric baseline, treatment and outcome these 4 groups mainly did not statistical differ from each other.But due to the sample size both groups were hardly comparable.

However, the majority of the questioned patients (n = 109) reported that the AB reduced pain after surgery, especially among patients who underwent laparoscopic IHR (p = 0.0428). These results confirm the assumptions of several authors that the AB has a pain reducing effect [12,13].

As negative effects, it is possible that the AB may induce atrophy due to the lack of using the abdominal muscles [12]. Moreover, Vailas et al. demonstrated that ambulation significantly enhances the healing process of tissue compared to the tissue in an immobilized state [17]. During our survey, at least 50 of 163 questioned patients reported that the AB induced immobility. In contrast, Cheifetz et al. (2010) performed a RCT analyzing the usefulness of an AB following major abdominal surgery. The authors postulated that the AB may enhance the speed of postoperative recovery of walking and physical performance [18]. But these trials did not focus on incisional hernias like the publication at hand.

In conclusion, the evidence for using an AB following ventral hernia repair is very low. Positive effects are not proven yet. Moreover, negative effects are supposed in literature. The survey at hand revealed for the first time an AB induced immobility as a negative effect. Therefore, we assume that the prescription of an AB may not be necessary. The avoidance of an AB may reduce the individual incapacity of working and the time of immobility, and would therefore have a positive social-economic impact. However, the evidence level of a survey, naturally, is low. Due to a small number of questioned individuals and the study design, our results should not be over-interpreted. Two different types of ABs were prescribed among the two 2 survey conducting hospitals. With an average price of 30 euros for one AB, costs should not heavily impact hospital budgets in Western Europe.

### 4.2 Physical rest

Recommending physical rest as well as avoidance of lifting heavy weights has been part of the postoperative-treatment after hernia repair for a long time. Bassini already (1890) recommended a period of physical rest for 6 weeks after inguinal hernia repair [19]. The rationale for recommending physical rest is unclear, however, physical rest is commonly attributed to beliefs about wound healing and the possible benefits of a prolonged period of inactivity in order to prevent recurrences. The clinical impact of physical rest on the postoperative outcome following hernia repair is unclear. To that, Lichtenstein published the results of 6321 cases of herniorhaphies. Despite immediate resumption of activity postoperatively, a recurrence rate of only 0.7% was revealed [20]. In regards to IHR, no correlation was detected between prolonged physical rest and the recurrence rate after reviewing the literature and questioning surgical departments in Germany about their postoperative outcome following IHR [11]. Therefore, it is perceivable that shortened physical rest may have no negative impact on the postoperative results after IHR. To add more evidence on this topic, we conducted the survey at hand.

To further analyze the effect of a prolonged duration of physical rest, we differentiated the individuals into two groups (Group I (n = 33): postoperative duration of physical rest for 0–4 weeks, Group II (n = 46) 5 - >14 weeks). Concerning the postoperative incidence of a relapse, a seroma formation, and a pulmonary infection, no statistical significance was detected between the two groups. These findings may confirm our previous results that there is no correlation between prolonged physical rest and a rate of recurrence [11]. However, as mentioned above, the evidence level of a survey, naturally, is low. Due to a small number of individuals and the study design, our results should not be over-interpreted. Moreover, as far as the age and the surgical approach go, the two groups statistically differ from one other (Table 3).

We assume that a shortened period of physical rest can be recommended following IHR. This can reduce the time of immobility and would therefore have a positive social-economic impact. The publication by Vailas et al. demonstrated in an animal model that ambulation significantly enhances the healing process by inducing a rapid return of tissue DNA and collagen synthesis compared to the animal's tissue in an immobilized state, which would support our hypothesis. Furthermore, in literature, early ambulation and return to work is not described as being a risk factor for recurrence [3,5,6]. Additionally, Majercik performed a prospective animal study involving 12 female pigs. They received an IHR with a laparoscopic mesh placement. The animals were put to sleep at different time andthe strength of the tissue attachment to the mesh was measured. The authors revealed, that 74% of strength that was seen after 12 weeks was already achieved two weeks after surgery. The number of samples in the mentioned animal study was low, but it confirms our assumption, that a shortened period of physical rest after IHR should be recommended.

To further prove our mentioned assumptions concerning an unproven positive impact of an AB and a prolonged period of physical rest after IHR we univariate analyses our survey data among individuals with different load-depended work. The patients of group II, who perform heavy physical labor (Table 4) wore the AB and physical rested not statistical significant longer than the patients of group I (light physical labor). Concerning the surgical approach, no statistical significance was detected between the two groups (p = 0.7897). The patients of group II were even younger (p = 0.0185). In literature, physical rest and wearing an AB are advised to improve wound healing. One could postulate that the heavy workload of the individuals of group II leads to more recurrences and seroma formations. In contrast, the postoperative incidence of recurrences (p = 0.093) and seroma formations (p = 0.9999) did not statistically differ between the two groups after univariate analysis. Yet again, due to a small number of questioned individuals and the study design, our results should not be considered as significant.

As survey limitations concerning a larger sample size would determine more valuable date. In terms of biometric data's we asked about the present age, BMI and profession. Revealing this data at the time of operation would determine more valuable data. On the other hand, we focused on a time period of only five years. The revealed data's may be similar. The questionnaire neither did ask for the exact time of a possible recurrence, pulmonary infection, seroma formation nor for the exact level of pain reduction and movement limitation following surgery.

For a more valuable analysis the width of the treated hernia should be known. On the other hand at the partizipating hospitals the open procedure naturally was mostly performed after diagnosing a width around 7 cm. Therefore we assume that the open procedure group contains patients suffering from a more large IHR.

Analyzing the questionnaires revealed that the patient's frequent physical rest and wearing the AB are recommended. Apparently, some questions were not answered correctly because we never recommend 3, 5 and 12 weeks as postoperative treatment. It appears that some patients may not properly remember the recommendations or did not want to admit a noncompliant behavior. This would then decrease the value of our findings.

The majority of our patients were retired. The role of being on sick leave naturally plays a smaller role with these individuals. The main aim of investigations and definitions of postoperative treatment may be to shorten the period of being on sick leave. By reducing the individual incapacity for work and immobility, this would have a social-economic impact. Therefore, further clinical trials may focus on actual working patients.

Further studies should also analyze the impact of different postoperative recommendations on the rate of postoperative wound infections.

We did not use a hernia-related quality of life questionnaire like the ‘Carolina Comfort Scale (CCS)’. But in order to receive a high number of patients who would reply to questionnaires, we focused on a small number of simple questions.

## 5 Conclusions

Due to our findings, we assume that an AB may induce immobility but also may reduce postoperative pain. A prolonged period of physical rest does not seem to have an impact on the postoperative outcome following an IHR.

Therefore, a shortened duration of physical rest and wearing an AB following an IHR may be advised. This would have a social-economic impact in reducing the individual incapacity to work.

Further investigations with randomized clinical trials focusing on different durations of physical rest and the effect of wearing an AB including a long-term follow-up are mandatory to elevate our hypothesis.

## Ethical approval

Ethical approval received.

## Sources of funding

All authors have no source of funding.

## Author contribution

Dr. med. Christoph Paasch (corresponding author):

Contribution to the paper: author, data collection, data analysis and interpretation, writing the paper, examination and treatment of the patient.

Dr. Gianluca Desanto (co-author):

Contribution to the paper: data analysis, examination and treatment of the patient.

Fr. Katherina Böttge (co-author):

Contribution to the paper: data analysis, examination and treatment of the patient.

Dr. Ulrich Gauger (co-author):

Contribution to the paper: data analysis.

Dr. med. Stefan Anders (co-author):

Contribution to the paper: data analysis, examination and treatment of the patient.

Prof. Roland Croner (co-author):

Contribution to the paper: data analysis, examination and treatment of the patient.

Dr. med. Eric Lorenz (co-author):

Contribution to the paper: data analysis, examination and treatment of the patient.

Prof. Dr. Martin W. Strik (co-author):

Contribution to the paper: data analysis, examination and surgical treatment of the patient.

## Conflicts of interest

None.

## Research registry number

The study has been registerd in the german clinical trial registry (DRKS, Deutsches Register klinischer Studien; https://www.drks.de) with the ID DRKS00016634.

## Guarantor

Dr. med. Christoph Paasch.

## References

[1] Hoer J., Lawong G., Klinge U., Schumpelick V. (2002). Factors influencing the development of incisional hernia. A retrospective study of 2,983 laparotomy patients over a period of 10 years. Chirurg.

[2] Burger J.W., Luijendijk R.W., Hop W.C., Halm J.A., Verdaasdonk E.G., Jeekel J. (2004). Long-term follow-up of a randomized controlled trial of suture versus mesh repair of incisional hernia. Ann. Surg..

[3] Bittner R., Bingener-Tasey J., Dietz U., Fabian M., Ferzli G.S., Fortelny R.H., Kockerling F., Kukleta J., Leblanc K., Lomanto D., Misra M.C., Bansal V.K., Morales-Conde S., Ramshaw B., Reinpold W., Rim S., Rohr M., Schrittwieser R., Simon T., Smietanski M., Stechemesser B., Timoney M., Chowbey P., International Endohernia S. (2014). Guidelines for laparoscopic treatment of ventral and incisional abdominal wall hernias (International Endohernia Society (IEHS)-part 1. Surg. Endosc..

[4] Anthony T., Bergen P.C., Kim L.T., Henderson M., Fahey T., Rege R.V., Turnage R.H. (2000). Factors affecting recurrence following incisional herniorrhaphy. World J. Surg..

[5] Bittner R., Bingener-Tasey J., Dietz U., Fabian M., Ferzli G., Fortelny R., Kockerling F., Kukleta J., LeBlanc K., Lomanto D., Misra M., Morales-Conde S., Ramshaw B., Reinpold W., Rim S., Rohr M., Schrittwieser R., Simon T., Smietanski M., Stechemesser B., Timoney M., Chowbey P. (2014). Guidelines for laparoscopic treatment of ventral and incisional abdominal wall hernias (International Endohernia Society [IEHS])-Part III. Surg. Endosc..

[6] Bittner R., Bingener-Tasey J., Dietz U., Fabian M., Ferzli G.S., Fortelny R.H., Kockerling F., Kukleta J., LeBlanc K., Lomanto D., Misra M.C., Morales-Conde S., Ramshaw B., Reinpold W., Rim S., Rohr M., Schrittwieser R., Simon T., Smietanski M., Stechemesser B., Timoney M., Chowbey P. (2014). Guidelines for laparoscopic treatment of ventral and incisional abdominal wall hernias (International Endohernia Society [IEHS])-Part 2. Surg. Endosc..

[7] Schwarz J., Reinpold W., Bittner R. (2016). Endoscopic mini/less open sublay technique (EMILOS)-a new technique for ventral hernia repair. Langenbeck's Arch. Surg..

[8] Rives J., Pire J.C., Flament J.B., Palot J.P., Body C. (1985). Treatment of large eventrations. New therapeutic indications apropos of 322 cases. Chirurgie.

[9] Sajid M.S., Bokhari S.A., Mallick A.S., Cheek E., Baig M.K. (2009). Laparoscopic versus open repair of incisional/ventral hernia: a meta-analysis. Am. J. Surg..

[10] Sajid M.S., Leaver C., Baig M.K., Sains P. (2012). Systematic review and meta-analysis of the use of lightweight versus heavyweight mesh in open inguinal hernia repair. Br. J. Surg..

[11] Paasch C., Anders S., Strik M.W. (2018). Postoperative-treatment following open incisional hernia repair: a survey and a review of literature. Int. J. Surg..

[12] Bouvier A., Rat P., Drissi-Chbihi F., Bonnetain F., Lacaine F., Mariette C., Ortega-Deballon P., Pour La Federation de Recherche en C. (2014). Abdominal binders after laparotomy: review of the literature and French survey of policies. Hernia.

[13] Rothman J.P., Gunnarsson U., Bisgaard T. (2014). Abdominal binders may reduce pain and improve physical function after major abdominal surgery - a systematic review. Dan Med J.

[14] Agha R.A., Borrelli M.R., Vella-Baldacchino M., Thavayogan R., Orgill D.P., Group S. (2017). The STROCSS statement: strengthening the reporting of cohort studies in surgery. Int. J. Surg..

[15] Christoffersen M.W., Olsen B.H., Rosenberg J., Bisgaard T. (2015). Randomized clinical trial on the postoperative use of an abdominal binder after laparoscopic umbilical and epigastric hernia repair. Hernia.

[16] Kaafarani H.M., Hur K., Hirter A., Kim L.T., Thomas A., Berger D.H., Reda D., Itani K.M. (2009). Seroma in ventral incisional herniorrhaphy: incidence, predictors and outcome. Am. J. Surg..

[17] Vailas A.C., Tipton C.M., Matthes R.D., Gart M. (1981). Physical activity and its influence on the repair process of medial collateral ligaments. Connect. Tissue Res..

[18] Cheifetz O., Lucy S.D., Overend T.J., Crowe J. (2010). The effect of abdominal support on functional outcomes in patients following major abdominal surgery: a randomized controlled trial. Physiother. Can..

[19] Bassini E. (1890).

[20] Lichtenstein I.L. (1987). Herniorrhaphy. A personal experience with 6,321 cases. Am. J. Surg..

