# Supplementary material for: Patient reported outcome following incisional hernia repair: A survey on 163 patients at two maximum care hospitals
Source: Ann Med Surg (Lond). 2019 Jun 15;44:5–12. doi: 10.1016/j.amsu.2019.06.005 (PMC6586918; doi:10.1016/j.amsu.2019.06.005)
Supplement: Multimedia component 3 [file mmc3.docx]

| The STROCSS Guideline | | |
| --- | --- | --- |
| Item no. | **Item description** | Page Number |
| 1 | Title. The words “cohort” and the area of focus should appear in the title (e.g. disease, exposure/intervention or outcome). Whether the study is retrospective or prospective should also be stated. | 1 |
| 2a | Abstract - Introduction What is the background and scientific rationale for the research question. | 1 |
| 2b | Abstract - Methods - Describe the study design (cohort design, retrospective or prospective, single or multi-centre, etc), what was done to each group, how, when was it done and by whom. | 1 |
| 2c | Abstract - Results What was found. Give the results for the main outcomes. | 1 |
| 2d | Abstract - Conclusion - What have we learned and what does it mean. Where should future research go. | 1 |
| 3 | Explain the scientific background and rationale for the cohort study. What are objectives, research questions and the hypotheses. | 1-2 |
| 4a | Registration and ethics State the research registry number in accordance with the declaration of Helsinki - "Every research study involving human subjects must be registered in a publicly accessible database before recruitment of the first subject" (this can be obtained from; ResearchRegistry.com or ClinicalTrials.gov or ISRCTN). Even retrospective studies should be registered prior to submission. | 4 |
| 4b | Ethical Approval - State whether ethical approval was needed and if so, what the relevant judgement reference from the IRB or local ethics committee was? If ethical approval was not needed, state why. | 4 |
| 4c | Protocol - Was a research protocol developed apriori? Where can it be accessed. Was it published in a journal e.g. IJS Protocols, BMJ Open, etc, if so, provide the reference. | no |
| 5a | Study design - State the research is a cohort study and whether prospective or retrospective in design, whether single or multi-centre. | 2 |
| 5b | Setting - Describe the setting(s)and nature of the institution in which the patient was managed; academic, community or private practice setting? Location(s), and relevant dates, including periods of recruitment, exposure, follow-up, and data collection | 2-3 |
| 5c | Cohort Groups - State the number of groups in the study. What interventions will each group receive? | 2-4 |
| 5d | Sub-group – Analysis. Any planned sub-group analyses are specified / Describe any methods used to examine subgroups and interactions. | 3 |
| 6a | Participants - State any eligibility (inclusion/exclusion) criteria and the sources and methods of selection of participants. Describe length and methods of follow-up. | 2-4 |
| 6b | Recruitment - State the methods of how patients or participants were recruited to each group, over what time periods. | 2-4 |
| 6c | Sample size calculation Whether there was calculation of margin of error or a prior analysis to determine study population, or mention of how appropriate study sample was determined. | / |
| 7a | Pre-intervention considerations - e.g. Patient optimisation: measures taken prior to surgery or other intervention e.g. treating hypothermia/hypovolaemia/hypotension in burns patients, ICU care for sepsis, dealing with anticoagulation/other medications and so on. | / |
| 7b | Types of intervention(s) deployed - To include reasoning behind treatment offered (pharmacological, surgical, physiotherapy, psychological, preventive) and concurrent treatments (antibiotics, analgesia, anti-emetics, nil by mouth, VTE prophylaxis, etc). Medical devices should have manufacturer and model specifically mentioned. | / |
| 7c | Peri-intervention considerations - Administration of intervention (what, where, when and how was it done, including details for surgery; anaesthesia, patient position, use of tourniquet and other relevant equipment, preparation used, sutures, devices, surgical stage (1 or 2 stage, etc) and operative time. Pharmacological therapies should include formulation, dosage, strength, route and duration). Authors are encouraged to use figures, diagrams, photos, video and other multimedia to explain their intervention. | / |
| 7d | Who performed the procedure(s) - Operator experience for each group (position on the learning curve for the technique if established, specialisation and prior relevant training). | 2 |
| 7e | Quality control - What measures were taken to reduce inter or intra-operator variation. What measures were taken to ensure quality and consistency in the delivery of the intervention e.g. independent observers, lymph node counts, etc | 2-4 |
| 7f | Post-intervention considerations - e.g. post-operative instructions and place of care. Important follow-up measures - diagnostic and other test results. Future surveillance requirements - e.g. imaging surveillance of endovascular aneurysm repair (EVAR) or clinical exam/ultrasound of regional lymph nodes for skin cancer. | 4-7 |
| 8 | Outcomes - What primary and secondary (if any) outcomes will be assessed and how are they defined. Definitions should be clear and precise. Appropriate references to validation of outcome measures used should be provided if they exist. | 4-7 |
| 9 | Statistical methods - Clearly outlined statistical tests used to compare the outcomes between an intervention group and a comparison group, state whether pre-existing differences and known confounders were controlled.  The statistical package used should be mentioned. | 3 |
| 10a | Participants recruited with a flow diagram - Report numbers involved in each group and use a flow diagram to show recruitment, non-participation, cross-over, withdrawal from the study with reasons. | / |
| 10b | Comparison between groups including a table - Provide a table comparing the demographic, clinical/prognostic features (co-morbidities, tumour staging, smoking status, etc) and relevant socioeconomic characteristics of each group and whether numerical differences are significant (using p-values and/or confidence intervals as appropriate). Were the groups matched and if so, how. | 4-7  Table  1-4 |
| 10c | Changes - Any changes in the interventions during the course of the study (how has it evolved, been altered or tinkered with, what learning occurred, etc) together with rationale and a diagram if appropriate. Degree of novelty for a surgical technique/device should be mentioned and a comment on learning curves should be made for new techniques/devices. | / |
| 11a | Outcomes and follow-up - Clinician assessed and patient-reported outcomes (when appropriate) should be stated for each group (size of effect with raw numbers and percentages) with inclusion of the time periods at which assessed. Relevant photographs/radiological images should be provided e.g. 12-month follow-up.Make it clear which confounders were adjusted for and which were not. | 4-7 |
| 11b | Intervention adherence/compliance and tolerability - How was this assessed. Describe loss to follow-up (express as a percentage and a fraction) or cross-over between group and any explanations for them. | / |
| 11c | Complications and adverse or unanticipated events - Described in detail and ideally categorised in accordance with the Clavien-Dindo Classification. How they were prevented, mitigated, diagnosed and managed. Blood loss, wound complications, re-exploration/revision surgery, 30-day post-op and long-term morbidity/mortality may need to be specified. | / |
| 12 | Summarise key results | 10 |
| 13 | Discussion of the relevance of the findings and rationale for conclusions - Relevant literature, implications for clinical practice guidelines, how have the indications for a new technique/device been refined and how do outcomes compare with established therapies and the prevailing gold standard should one exist and any relevant hypothesis generation. The rationale for any conclusions. | 8-10 |
| 14 | Strengths and limitations of the study | 10 |
| 15 | State what needs to be done next, further research with what study design(s). | 10 |
| 16 | State the key conclusions from the study and key directions for future research | 10 |
| 17a | State any conflicts of interest | 11 |
| 17b | State any sources of funding | 11 |
